# The Venom of the Spine-Bellied Sea Snake (*Hydrophis curtus*): Proteome, Toxin Diversity and Intraspecific Variation

**DOI:** 10.3390/ijms18122695

**Published:** 2017-12-12

**Authors:** Vanessa Neale, Javier Sotillo, Jamie E. Seymour, David Wilson

**Affiliations:** 1 College of Public Health, Medical and Veterinary Sciences, James Cook University, McGregor Road, Smithfield, Cairns 4878, Australia; 2 Australian Institute of Tropical Health and Medicine (AITHM) and Centre for Biodiscovery and Molecular Development of Therapeutics (CBMDT), James Cook University, McGregor Road, Smithfield, Cairns 4878, Australia; javier.sotillo@jcu.edu.au (J.S.); jamie.seymour@jcu.edu.au (J.E.S.)

**Keywords:** *Hydrophis curtus*, spine-bellied sea snake, sea snake venom, venom proteome, venomics, intraspecific variation

## Abstract

The spine-bellied sea snake (*Hydrophis curtus*) is known to cause human deaths, yet its venom composition has not yet been proteomically characterised. An in-depth proteomic analysis was performed on *H. curtus* venom from two different seasons, January and June, corresponding to adults and subadults, respectively. Venoms from adult and subadult *H. curtus* individuals were compared using reversed-phase high-performance liquid chromatography (RP-HPLC), matrix-assisted laser desorption ionisation-time of flight (MALDI-TOF) mass spectrometry and liquid chromatography electrospray ionisation mass spectrometry (LC-ESI-MS) to detect intraspecific variation, and the molecular weight data obtained with ESI-MS were used to assess toxin diversity. RP-HPLC and LC-ESI-MS/MS were used to characterise the venom proteome and estimate the relative abundances of protein families present. The most abundant protein family in January and June venoms is phospholipase A_2_ (PLA_2_: January 66.7%; June 54.5%), followed by three-finger toxins (3FTx: January 30.4%; June 40.4%) and a minor component of cysteine-rich secretory proteins (CRISP: January 2.5%; June 5%). Trace amounts of snake venom metalloproteinases (SVMP), C-type lectins and housekeeping and regulatory proteins were also found. Although the complexity of the venom is low by number of families present, each family contained a more diverse set of isoforms than previously reported, a finding that may have implications for the development of next-generation sea snake antivenoms. Intraspecific variability was shown to be minor with one obvious exception of a 14,157-Da protein that was present in some January (adult) venoms, but not at all in June (subadult) venoms. There is also a greater abundance of short-chain neurotoxins in June (subadult) venom compared with January (adult) venom. These differences potentially indicate the presence of seasonal, ontogenetic or sexual variation in *H. curtus* venom.

## 1. Introduction

The viviparous sea snakes (Elapidae: Hydrophiinae: Hydrophinii) consist of 62 species that are phylogenetically placed within the terrestrial elapids [1]. Phylogenetic relationships among sea snakes have been clarified in recent years, with *Hydrophis* and *Aipysurus* emerging as the two major clades [1]. Sea snakes are widespread in the shallow tropical and subtropical marine waters of northern Australia, the western Pacific and Indian Oceans and the Persian Gulf [2,3], feeding predominantly on sedentary, bottom-dwelling fish [4]. Two or three are generalist feeders, whereas most are specialists, favouring prey such as eels, gobies and catfish [4].

Sea snake venoms are relatively simple mixtures, containing a limited number of toxin families and isoforms [5,6], potentially as a result of their limited fish diet, which is believed to require a minimal suite of toxins [5]. Characteristically, the venom is dominated by toxins from two protein families, the neurotoxic three-finger toxins (3FTx) and myotoxic phospholipase A_2_ (PLA_2_) [6,7,8,9]. Sea snake venom can be dangerous to humans, in severe cases causing myolysis and/or neuromuscular paralysis that can be lethal without prompt treatment [10,11,12,13]. Most at risk are Asian fishermen using traditional methods, who encounter the animals when handling nets or sorting fish [12]. The spine-bellied sea snake (*Hydrophis curtus*; synonymous with *Lapemis curtus*, *Lapemis hardwickii* and *Hydrophis hardwickii*) [14], the most generalist feeder of all the sea snakes [4], is one of the species known to be responsible for human fatalities [15,16]. It is all the more hazardous because it is likely to be encountered by people, being one of the most widely-distributed sea snakes, found from northern Australia to the Persian Gulf via Southeast Asia [14], and is common throughout much of its range [12,15,17]. Further, it is prone to biting defensively if captured, such as if caught in a fishing net [18].

The degree of intraspecific venom variation in sea snakes is unclear as little work has been done and the available evidence is contradictory. In *H. curtus* venom, liquid chromatography electrospray ionisation mass spectrometry (LC-ESI-MS) shows some differences in the molecular weights of venom proteins between widely-separated populations [5], suggesting compositional variation may exist. Conversely, studies on other species comparing reversed-phase high-performance liquid chromatography (RP-HPLC) chromatograms show limited variation, even between allopatric populations [6,8]. Fourteen toxins in total have been isolated and sequenced from *H. curtus* venom. There are four PLA_2_ toxins (Q8UW30, Q8UW08, A3FM57 and Q8UW31) [19,20], three 3FTx short-chain α-neurotoxins (P68416, Q8UW26 and Q8UW27) [21,22] and three 3FTx long-chain α-neurotoxins (Q8UW28, A3FM53 and Q8UW29) [19]. Two cysteine-rich secretory proteins (CRISP) (Q8UW25 and Q8UW11) [23], a snake venom serine protease (SVSP) (Q5MCS0) [24] and a C-type lectin (A3FM55) have also been found [19]. In accordance with the presence of 3FTx, *H. curtus* venom shows post-synaptic neurotoxicity in chick biventer cervicis nerve-muscle preparations [25]. The venom demonstrates myotoxicity in mice [26], consistent with the presence of PLA_2_ toxins, and shows only low levels of other enzymatic activity [27]. An estimate of the relative proportions of toxin families in *H. curtus* venom was attempted using cDNA cloned from venom gland mRNA, suggesting the venom contained 43.4% 3FTx, 9.8% PLA_2_ and 2.3% CRISP [28].

The composition of *H. curtus* venom has not yet been comprehensively determined with proteomic analysis. In fact, the venom proteomes of only four sea snakes have been published so far, despite the group’s medical significance [6,7,8,9]. To increase understanding of sea snake venoms, and *H. curtus* venom specifically, this study presents a proteomic analysis of pooled *H. curtus* venom from two different seasons, January and June, corresponding to adults and subadults, respectively. RP-HPLC and LC-ESI-MS/MS were employed to characterise the venom proteome and estimate the relative abundances of protein families present using a modified version of the “snake venomics” workflow proposed by Calvete [29]. Additionally, intraspecific venom variation was assessed with RP-HPLC, matrix-assisted laser desorption ionisation-time of flight (MALDI-TOF) mass spectrometry and LC-ESI-MS, and toxin diversity was assessed using the data obtained with LC-ESI-MS.

## 2. Results

### 2.1. Assessment of Variation in H. curtus Venom

**Figure 1:**
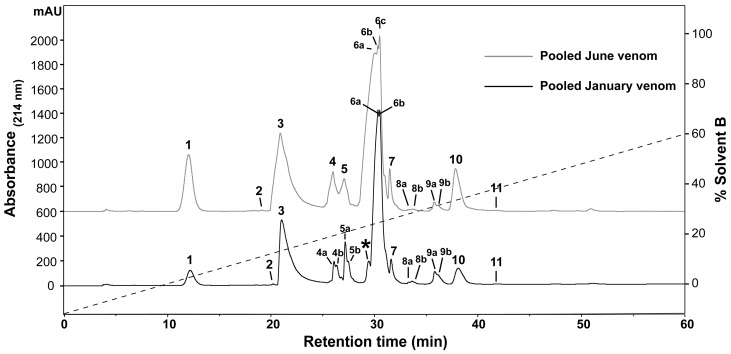
Reversed-phase high-performance liquid chromatography (RP-HPLC) chromatogram comparison of pooled January and June *Hydrophis curtus* venoms. Two groups of samples were collected from wild-caught snakes in January and June of 2016 and pooled separately. Pooled samples were separated by RP-HPLC (Phenomenex Jupiter C_4_, 4.6 mm × 250 mm, 5 µm, 300 Å) (Phenomenex, Torrance, CA, USA) and monitored at 214-nm absorbance to compare protein and peptide components. January venom has three extra peaks, 4b, 5b and the peak marked with an asterisk (*). June venom has one peak not present in January venom (6c). The dashed line indicates the solvent gradient from 0–60% Solvent B.

**Figure 2:**
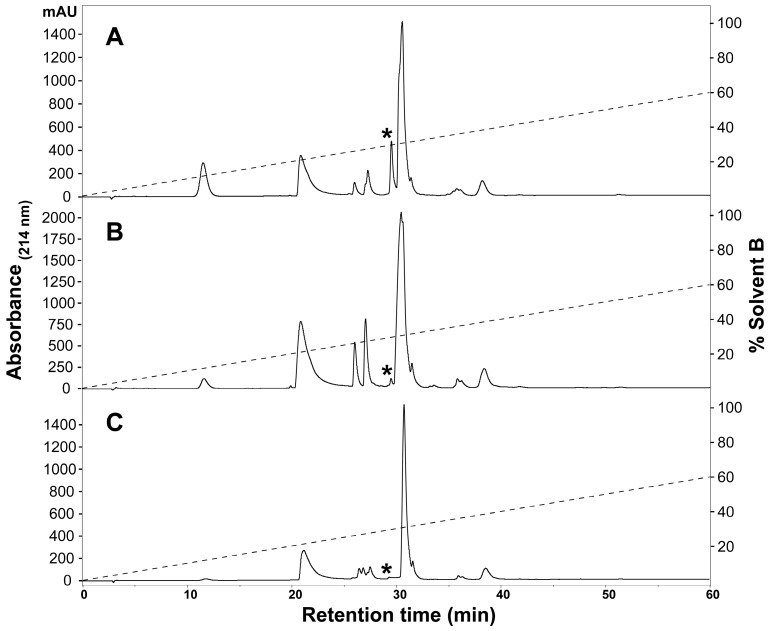
Representative individual *Hydrophis curtus* venom samples from January 2016, highlighting the variable 14-kDa peak. RP-HPLC (Phenomenex Jupiter C_4_, 4.6 mm × 250 mm, 5 µm, 300 Å) (Phenomenex, Torrance, CA, USA) chromatograms of (**A**) Sample 11, (**B**) Sample 9 and (**C**) Sample 5 are shown and were monitored at 214-nm absorbance. The **asterisks** point to a peak containing a molecular weight of 14,157 Da that is highly variable in its relative abundance and presence. The dashed lines indicate the solvent gradient from 0–60% Solvent B.

**Figure 3:**
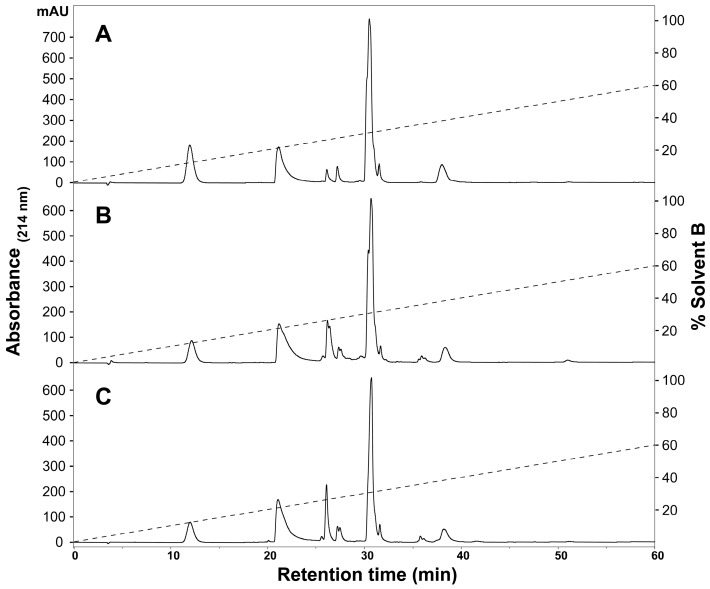
Representative individual *Hydrophis curtus* venom samples from June 2016. RP-HPLC (Phenomenex Jupiter C_4_, 4.6 mm × 250 mm, 5 µm, 300 Å) (Phenomenex, Torrance, CA, USA) chromatograms of (**A**) Sample 7, (**B**) Sample 10 and (**C**) Sample 2 are shown and were monitored at 214-nm absorbance. The dashed lines indicate the solvent gradient from 0–60% Solvent B.

To determine the presence of any intraspecific variation present in *H. curtus* venom, the venom composition was analysed and compared by RP-HPLC and LC-MS. An initial comparison of the same venom sample by RP-HPLC using a C_4_ and a C_12_ column showed improved peak resolution with the C_4_ column. A comparison of RP-HPLC chromatograms from January and June pooled samples shows most peaks are common (Figure 1). January venom has three extra peaks, 4b, 5b and the peak marked with an asterisk (*). June venom has one peak not present in January venom (6c). ESI-MS molecular weight data were generally congruent between June Peak 4 and January Peaks 4a and 4b; June Peak 5 and January Peaks 5a and 5b; and June Peaks 6a–6c and January Peaks 6a–6b (Table 1). In most of the exceptions, the molecular weights were detected in neighbouring peaks of the other venom or were present in small quantities, with the exception of a molecule 13,336 Da in size that was abundant in Peak 4a of the January pool. The peak marked with an asterisk (*), which corresponds to 2.5% of the total peak area of the RP-HPLC chromatogram, predominantly consists of a molecule 14,157 Da in size. In individual January adults, this peak varies in abundance and presence. In a comparison of three representative individuals (Figure 2A–C), the peak is present and comparatively abundant in Sample 11 at 7.2% of total peak area (Figure 2A), reduced in Sample 9 to 0.7% of total peak area (Figure 2B) and virtually non-existent in Sample 5 (Figure 2C). This molecule and the peak are absent in all June venoms (Figure 3A–C), including the single adult sample available.

**Figure 4:**
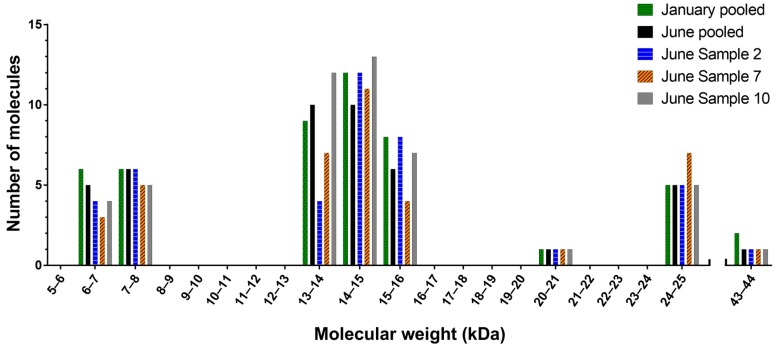
Histogram of the mass landscape of *Hydrophis curtus* venom. Highlight of the abundance, variation and distribution of the molecular weights in the January and June pooled venom samples and three representative June individuals. Molecular weights are divided into 1-kDa molecular weight bins.

Analysis of January individuals by MALDI-TOF mass spectrometry showed that molecular weight data were consistent between individuals, with very few exceptions. A comparison of MALDI-TOF and ESI-MS showed that the chromatography resolution and molecular weight data were consistent between the two (see Figure S1 in Supplementary Materials S9), except that more masses were detected with ESI-MS. January and June pooled samples, and all individual June samples were therefore analysed by ESI-MS. Figure 4 shows the number, distribution and variation of molecules present in the pooled *H. curtus* samples and three representative June individuals. It depicts three discrete clusters of components with molecular weights ranging from 6–8 kDa, 13–16 kDa and 24–25 kDa, which are consistent with the 3FTx, PLA_2_ and CRISP protein families, respectively [29]. One or two molecular weights are also found in the 20–21 kDa and 43–44 kDa ranges. The January and June pools contained 49 and 44 unique molecules respectively. Of those, 37 were common to both samples, leaving 12 unique molecules in the January pool and seven in the June pool. There was more variation between individuals, with the number of molecules found in each sample ranging from 26–48. Individual venoms, while showing a high degree of consistency between the major components of each peak, did not contain all molecules, and in some cases, molecules were unique to a single sample. Variation in the number of molecules between the representative individual venoms is particularly obvious in Bins 13–14 and 15–16 (Figure 4). In the June pool, the proportion of molecules in the 6–8 kDa cluster (3FTx) accounted for 25.0% of the total versus 59.1% for the 13–16 kDa cluster (PLA_2_). The breakdown of the remainder was 11.4% for 24–25 kDa (CRISP) and 2.3% for both 20–21 kDa and 43–44 kDa. The almost identical January pool breakdown of molecular weights was as follows: 6–8 kDa 24.5%; 13–16 kDa 59.2%; 20–21 kDa 2.0%; 24–25 kDa 10.2%; and 43–44 kDa 4.1%.

### 2.2. Protein Identification with ProteinPilot and MASCOT

Searches were conducted using ProteinPilot and MASCOT, and the full results are included in Supplementary Tables S1–S8, including MS/MS-derived sequences, mass to charge ratios (*m*/*z*) and charges (*z*) from ProteinPilot (Tables S1–S4). Table 1 summarises the results from the ProteinPilot searches of all peaks in the June pooled venom (Table S2) and the peak unique to January venom (Table S1) against the UniProtKB Serpentes database, as well as the molecular weights detected in ESI-MS analysis of January and June pooled venoms. 3FTx toxins are dominant in Peaks 1–3, PLA_2_ in Peaks 4–9b and CRISP in Peak 10. ESI-MS-derived molecular weights consistent with those families follow the same pattern. The phosphatidylethanolamine-binding protein 4 (U3FZ77) found in Peak 9b is consistent with the 20,615-Da molecule found in the same peak. A C-type lectin and a number of housekeeping and regulatory proteins are found in Peak 11, and SVMP is found in Peaks 4–5. This distribution of protein families is consistent with all other searches performed (Supplementary Tables S1–S8).

### 2.3. Relative Protein Abundance Estimations Using MASCOT emPAI

**Figure 5:**
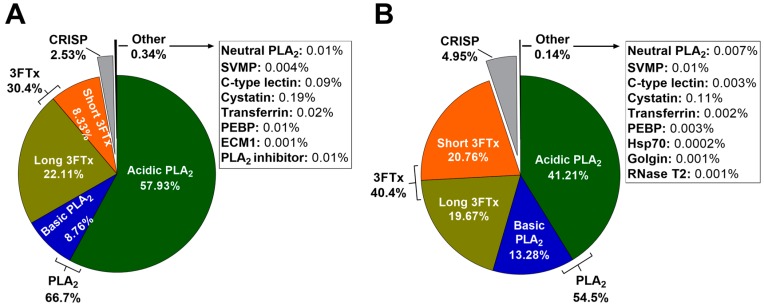
Composition of *Hydrophis curtus* venom by protein family for (**A**) January (adult) and (**B**) June (subadult) pooled venoms, as calculated by MASCOT exponentially-modified protein abundance index (emPAI) score. The pie charts show the relative abundances of phospholipase A_2_ (PLA_2_) (acidic, basic and neutral), three-finger toxins (3FTx) (long-chain and short-chain), cysteine-rich secretory protein (CRISP), snake venom metalloproteinase (SVMP), C-type lectins, cystatin, transferrin, phosphatidylethanolamine-binding protein (PEBP), heat shock protein (Hsp70), extra-cellular matrix protein (ECM1), PLA_2_ inhibitor, golgin and ribonuclease T2 (RNase T2).

Figure 5 shows the relative abundances of toxin families in pooled January (adult) and June (subadult) *H. curtus* venoms, which were calculated with MASCOT exponentially-modified protein abundance index (emPAI) scores from searches against the UniProtKB Serpentes database. In both January and June venoms, PLA_2_ is most abundant. In January venom, the total PLA_2_ is 66.7% (57.93% acidic PLA_2_ and 8.76% basic PLA_2_), while in June venom, the total PLA_2_ makes up a smaller proportion of the venom at 54.5% (41.21% acidic PLA_2_ and 13.28% basic PLA_2_). The acidic portion is composed almost entirely of one toxin, acidic phospholipase A_2_ 57 (Q8UW31), making it the most abundant toxin in the venom. 3FTx makes up most of the remainder in both venoms. January venom contains a total of 30.4% 3FTx (22.11% long-chain neurotoxins and 8.33% short-chain neurotoxins), while the proportion of 3FTx in June venom is higher at 40.4%, which is largely due to a greater proportion of short-chain neurotoxins (20.76%). The proportion of long-chain neurotoxins is similar between samples (19.67% and 22.11% in June and January venoms, respectively). CRISP takes up 2.53% and 4.95% in January and June venom respectively. Other components, including SVMP, C-type lectins and a handful of house-keeping and regulatory proteins, are present in trace amounts.

## 3. Discussion

### 3.1. Assessment of Variation in H. curtus Venom

The present study provides the first proteomic characterisation of the *H. curtus* venom, including venoms from two distinct seasons, January and June, which correspond to adults and subadults, respectively. This allowed us to assess not only the individual variation in venom, but also if seasonal and/or ontogenetic variation were present. Individual variation in toxin expression occurs regularly in snakes [30], as does ontogenetic variation, which is often driven by a change in diet as the snake matures to adulthood [31,32,33,34]; however, little evidence exists for seasonal variation [30]. In this sense, variations of all kinds are a highly relevant consideration when producing antivenoms in order to ensure all potential toxin activities can be neutralised [35,36].

The minimal variation observed in the *H. curtus* venom tested was expected since all the samples tested were collected within metres of each other from a sympatric population and there is no known driver for venom variation in viviparous sea snakes such as ontogenetic or seasonal diet changes [4]. Comparisons of RP-HPLC chromatograms for individuals and pooled samples showed that the venom was in fact remarkably similar. Despite that, one distinct difference emerged: the peak containing a 14,157-Da protein, which was found in some adult January animals. Since it was entirely absent from the subadult animals, which were all milked in June, it is possible that seasonal or ontogenetic variation is responsible for the difference. If June adults contain the protein and June subadults do not, it would be evidence for ontogenetic rather than seasonal variation. However, as the single adult June venom sample did not contain the protein, the question remains open. Because the protein only appears in some adults, a third possibility is that *H. curtus* has sexual venom variation between adult males and females. There is only a small amount of evidence of sexual venom variation in snakes [30,37,38], and whether that is the case here remains to be investigated. A fourth possibility is that this is merely a case of the individual variability typically found in snake venoms [30]. Whether or not the difference is practically important will depend on how the 14,157-Da protein affects venom activity. The size of the 14,157-Da protein is consistent with PLA_2_, and nothing other than 3FTx, CRISP or PLA_2_ was identified in that peak (Table 1). If the protein behaves similarly to other *H. curtus* PLA_2_ toxins, the practical effect of the difference may be minimal, in both an ecological and clinical setting. Further research into the reason for the difference and the activity of the protein is required. The variations that were found in MALDI-TOF mass spectrometry, ESI-MS and RP-HPLC results underscore the need to use a large and diverse pool of individuals for antivenom production [36], even in species that typically show little variation such as sea snakes, especially when the effects of the variable proteins on humans are not understood.

### 3.2. Protein Identification and Relative Abundance Estimations with ProteinPilot and MASCOT

Protein identifications were consistent with previously sequenced *H. curtus* toxins. Of the 14 toxins previously identified, ProteinPilot consistently matched 10 (as the synonymous *Hydrophis hardwickii*) (Table 1). A further two, both short-chain neurotoxins (Q8UW26 and Q8UW27), were regularly listed as matches of lower significance or less commonly as equal winner proteins, across all ProteinPilot searches (Supplementary Tables S1–S4). This is likely because both proteins are almost identical to the short-chain neurotoxin that was identified (P68416), with only one amino acid different, and ProteinPilot lacked the identifying peptide required to distinguish between them. A PLA_2_ (A3FM57) was listed as a match of lower significance once, in Peak 5 of the ProteinPilot search against the UniProtKB database of June venom (Supplementary Table S2). Only the serine protease harobin (Q5MCS0) was not identified at all. Proteins from a number of other snake species were matched, predominantly Australian terrestrial elapids (Table 1, Supplementary Tables S1–S8).

ESI-MS detected molecular weights similar to nine of the 14 known *H. curtus* toxins, as calculated with the ExPASy pI/*M*w calculator from known sequence data (Table 1). Those included all three short-chain neurotoxins, providing further support for their presence. ESI-MS did not detect the long-chain neurotoxins (A3FM53 and Q8UW29) and the C-type lectin (A3FM55) that were identified by ProteinPilot. It also did not detect the PLA_2_ (A3FM57) or the serine protease harobin (Q5MCS0); nor did ESI-MS detect molecular weights consistent with SVMP or most house-keeping proteins. Possible reasons for the failed detections in protein identification software and ESI-MS could be because the proteins are present only in trace quantities or are absent or different in Australian *H. curtus* venom compared with the Asian *H. curtus* specimens usually studied. Additionally, if the protein were identified from venom gland mRNA, as was the case for serine protease harobin (Q5MCS0) [24], it may not be readily expressed in the proteome.

The MASCOT emPAI protocol was employed to calculate the relative abundances of protein families in *H. curtus* venom. The emPAI score is proportional to the amount of an individual protein present in a mixed sample and has been found to produce equivalent results to protein staining methods [39]. The proteomic characterisation of *H. curtus* venom by LC-ESI-MS/MS, ProteinPilot and MASCOT confirmed that the protein families 3FTx and PLA_2_ dominate the venom (Table 1, Supplementary Tables S1–S8), continuing the trend found in all other sea snake venoms studied so far [6,7,8,9]. In *H. curtus*, PLA_2_ was the most abundant protein family, contrasting with *Hydrophis cyanocinctus*, *Hydrophis schistosus* and *Hydrophis platurus*, in which 3FTx is dominant [7,8,9]. PLA_2_ dominance of the venom is a distinction *H. curtus* shares with only one other sea snake that has been proteomically characterised, the olive sea snake (*Aipysurus laevis*) [6]. Until the *A. laevis* venom was characterised, 3FTx appeared to be dominant in the majority of sea snake venoms [7,8,9], even in *H. curtus*, where a previous investigation indicated that the venom included 43.4% 3FTx and 9.8% PLA_2_ [28]. However, this study was based on cDNA libraries cloned from venom gland mRNA, and the results might reflect a bias towards 3FTx mRNA in the cDNA method used or indicate that proteins are not translated from mRNA in a directly proportional manner [40].

The apparent dominance of 3FTx in the majority of sea snakes suggests that sea snake venoms should be neurotoxic to humans, as elapid short- and long-chain α-neurotoxins cause respiratory paralysis by binding to skeletal muscle nicotinic acetylcholine receptors (nAChRs) and, in the case of long-chain α-neurotoxins, neuronal α7 nAChR [41]. However, sea snake envenomations in humans are predominantly myotoxic, less often neurotoxic and rarely both [11,42]. It has been suggested that some short-chain sea snake neurotoxins are not as effective on humans due to an amino acid difference in the neuromuscular nAChR [43]. The results of the present study suggest an additional reason for human clinical presentation is interspecific, and possibly intraspecific, variations in the proportions of the two dominant components and, at a finer scale, the proportions of acidic and basic PLA_2_ components. PLA_2_ causes the breakdown of skeletal myofilaments [44,45] by inducing cellular degeneration with an influx of Ca^2+^ ions [46]. Some basic PLA_2_ toxins are more effective at penetrating the phospholipid bilayer than acidic PLA_2_ toxins [47,48], suggesting that a venom with a higher proportion of basic PLA_2_ would be a more effective myotoxin. The presence and proportion of basic and acidic PLA_2_ unquestionably differs between sea snakes: *H. curtus*, *H. schistosus* and *H. platurus* have both types [8,9], whereas all *A. laevis* PLA_2_s appear to be acidic [6]. Therefore, in sea snake envenomations, the effective proportion of either dominant protein family may be entirely different from the actual proportion. It would be useful to conduct an epidemiological study of worldwide sea snake envenomations to see if there is a correlation between the clinical effects of envenomation and the dominant toxin families or their subtypes in the species responsible.

Ecologically, it is unclear why the most dominant protein family alternates between 3FTx and PLA_2_ in different sea snake species. Nearly all sea snakes are teleost feeders [4], a comparatively limited diet that could result in sea snakes employing similar proportions of toxin families, yet this is not the case. It would be interesting to see if there is a connection between the proportions of toxin families and subtypes that make up the venom and dietary parameters such as size and type, as well as the degree of variation in prey eaten. Notably, there is a greater proportion of short-chain 3FTx and a corresponding decrease in the proportion of acidic PLA_2_ in the June subadult venom pool compared with the January adult venom pool (Figure 5). As with the 14,157-Da protein, this may indicate seasonal, sexual or ontogenetic variation, but it is hard to know if there is any ecological or clinical significance to this difference without further investigation. It would be worth analysing the venom of neonate *H. curtus* snakes to see if there is an even more distinct difference with the adult venom pool and/or investigating the lifetime diet of *H. curtus* to detect any change that might indicate seasonal or ontogenetic changes in venom. It has been suggested that PLA_2_ in sea snakes has a digestive role [8]. If so, it is possible that adult snakes could require more PLA_2_ to digest larger prey, whereas for small snakes, the ability to quickly immobilise prey with neurotoxins might be more important. A lower abundance of PLA_2_ in the venom of juveniles of some other elapid species when compared with the adult venoms has been previously observed, such as the Australian *Pseudonaja textilis* and *Pseudonaja affinis* [49] and the Asian *Naja atra* and *Naja kaouthia* [50,51]. Together with the findings in the present study, this may indicate a trend across the elapid group.

### 3.3. Toxin Diversity in H. curtus Venom

Although *H. curtus* venom was simple by the number of toxins families, it was unexpectedly diverse by the number of unique molecules. The ESI-MS data in Table 1 list a large number of unique molecular weights that did not match any known *H. curtus* toxin. This contrasts with a study that identified 13 molecular weights in Australian *H. curtus* venom using LC-ESI-MS [5], compared with 49 for pooled January venom and 44 for pooled June venom in the present study. The discrepancy is likely because the original study was conducted 14 years ago in 2003 when LC-ESI-MS was much less sensitive. Molecular weights consistent with 3FTx, PLA_2_ and CRISP toxin families were found in both studies, so the number of toxin families in *H. curtus* venom likely remains the same. However, the strong likelihood is that there are a number of novel isoforms within those families, particularly in PLA_2_, which makes up 59% of the number of unique molecules for both January and June pools. Furthermore, ProteinPilot identified <35% (at 95% confidence) of total MS/MS spectra in each RP-HPLC peak, and MASCOT was also unable to assign a large number of peptides to a match. This implies that there are proteins in the venom that have no homologs in either the NCBI or UniProtKB Serpentes databases, despite the comprehensive nature of both. Finally, in the protein identification results, there were matches to several toxins from venomous terrestrial elapids, suggesting that some sequences in *H. curtus* toxins are different from known toxins of any sea snake species. In this regard, a reference genome or a transcriptomic study will help in the identification of novel molecules present in the venom.

The possibility that *H. curtus* venom is more biochemically complex than currently understood has implications for sea snake antivenom manufacture and development and calls into question the idea that sea snakes possess minimal isoform diversity. Previous studies have shown limited diversity in both the number of families and diversity of isoforms, especially when compared with land snakes [5,6,7,9]. In *H. curtus*, however, toxin diversity is comparable to some terrestrial elapids such as the African *Naja* spp., where 3FTx and PLA_2_ also dominate and toxin numbers range from 22–32 per species [52], and the Australian-Papuan *Oxyuranus* spp. [53]. The limited diet of sea snakes is hypothesised to require only a minimal suite of toxins [5]. It is possible that *H. curtus* is an exception to the rule as it is a generalist fish feeder [4] and therefore requires a greater range of toxins to subdue prey. Certainly, the 2003 study [5] found that Australian *H. curtus* venom had the highest number of toxins of any sea snake tested except for Malaysian *H. curtus* venom, which had 14. However, in that same study, *H. schistosus*, which is a cat fish specialist [4], had an equivalent number of toxins to Australian *H. curtus* venom. This may contradict the hypothesis that *H. curtus*, as a generalist fish-feeder, possesses more toxin diversity, but further investigation is needed.

An updated LC-ESI-MS analysis of other sea snake venoms would give an indication of the amount of toxin diversity present in individual species and across the entire group, but to completely characterise the diversity of toxins in *H. curtus* and to verify that molecular weights detected by ESI-MS are proteins with a unique amino acid sequence, sequences need to be confirmed. Determining the extent of toxin diversity in sea snakes is important because if it is greater than currently believed, efforts to develop a new sea snake antivenom based on human monoclonal antibodies or peptide-based inhibitors as previously suggested [6] may be more complex than expected. However, the degree of complexity will also depend on how different the toxins in sea snake venom are, by sequence or activity, and what role they play in a human envenomation.

In conclusion, this study has provided insights into the proteomic composition of *H. curtus* venom. It also indicated the presence of venom variation, which may be due to sexual, seasonal or ontogenetic differences. We hope that further research will be undertaken to clarify the questions raised about the ecological and clinical implications of our findings.

## 4. Materials and Methods

### 4.1. Venom Samples

*Hydrophis curtus* venom was collected from wild individuals caught at Hey Point, Weipa (12°43203246.6″ S 141°53′34.4″ E) in the Gulf of Carpentaria, Australia, on two separate field trips. The first samples were collected from 11 adult specimens in January 2016. Samples from one adult and 10 subadults were collected in June 2016, where “subadult” is defined as an individual < 400 mm in total length and “adult” is an individual > 600 mm in total length. The sexes of the snakes are unknown. All venom samples were stored in liquid nitrogen during transport, then lyophilised and stored at −80 °C. Approval to collect venom from wild sea snakes was granted by the James Cook University Experimentation Ethics Review Committee under Approval Number A1575, September 2010.

### 4.2. Pooling Venom Samples

All samples of lyophilised *H. curtus* venom, except for the single June adult, were resuspended separately in 500 µL of Milli-Q (MQ; Merck Millipore, Bayswater, Victoria, Australia) water and pooled into their respective group, January or June. Thirty microliter of each sample were set aside to test for intraspecific variation. Pooled venom was separated into 100-µL aliquots, lyophilised and stored at −80 °C. Venom was pooled in order to investigate the average venom composition of the population at the respective time point.

### 4.3. Venom Fractionation and Comparative Profiling with Reversed-Phase HPLC

RP-HPLC was performed on all individual samples and on the January and June pools, which were resuspended in 30 µL of MQ water. Using an Agilent 1260 HPLC (Agilent, Santa Clara, CA, USA), a total of 20 and 25 µL from the individual and pooled samples respectively was injected into a C_4_ column (Phenomenex Jupiter, 4.6 mm × 250 mm, 5 µm particle diameter, pore size 300 Å) (Phenomenex, Torrance, CA, USA), with a column oven temperature of 35 °C. The sample was eluted using a linear gradient of 0.05% trifluoroacetic acid (TFA; Auspep, Tullamarine, Victoria, Australia) in MQ water (Buffer A) and 90% acetonitrile (ACN; Sigma-Aldrich, St Louis, MO, USA) with 0.045% TFA in MQ water (Buffer B) (0–60% B over 60 min; 60–90% B over 5 min; 90% B for 10 min; and 90–0% B over 5 min) at 1 mL/min. Absorbance was monitored at 214-nm, and fractions for both pooled samples were collected automatically into a 96-well, 1.09-mL deep well plate (Axygen, Union City, CA, USA) in one-minute intervals. ChemStation C.01.03 software (Agilent, Santa Clara, CA, USA) was used to standardise the base lines and calculate peak areas for each chromatogram.

### 4.4. Venom ‘Fingerprint’ Analysis Using LC-MS

Venom fingerprint analysis of January individuals was performed using RP-HPLC and a SCIEX TOF/TOF™ 5800 MALDI mass spectrometer (SCIEX, Framingham, MA, USA), mixing 0.7 μL of sample with 0.7 μL α-cyano-4-hydroxycinnamic acid (CHCA; Sigma-Aldrich, St. Louis, MO, USA) matrix at 7.5 mg/mL in 50% ACN/0.1% TFA onto an Opti-TOF 384-well plate. Venom fingerprint analysis of the pooled venoms, the June subadult venoms and the single June adult venom was conducted using a Shimadzu LC-MS 2020 single quadrupole mass spectrometer coupled with a Shimadzu Prominence HPLC system (Shimadzu, Kyoto, Japan). Five microliter of each *H. curtus* sample were separated with a Phenomenex XB-C_18_ column (Phenomenex Aeris, 2.1 mm × 150 mm, 3.6-µm particle diameter, pore size 100 Å) (Phenomenex, Torrance, CA, USA) at a flow rate of 250 µL/min and using a linear gradient of 0.1% formic acid (FA; Sigma-Aldrich, St. Louis, MO, USA) in MQ water (Buffer A) and 90% ACN (Sigma-Aldrich, St. Louis, MO, USA) with 0.09% FA in MQ water (Buffer B) (0–60% B over 60 min; 60–90% B over 5 min; 90% B for 5 min; and 90–0% B over 5 min). ESI-MS data were collected in profile acquisition mode (positive) over a scan range of 250–2000 *m*/*z*. Molecular weights were reconstructed from ion series using the ‘multi-charged ion analysis’ module of the Shimadzu LabSolutions software (Version 5.80) (Shimadzu, Kyoto, Japan).

### 4.5. In-Solution Trypsin Digestion

Fractions in the pooled samples corresponding to RP-HPLC peaks were lyophilised and reconstituted in 10 µL of 55 mM ammonium bicarbonate, vortexed for 10 s and centrifuged for 2 min at 16,000× *g* (5415D Eppendorf centrifuge). Where a peak comprised more than one fraction, these were combined. Samples were reduced with 2 mM 1,4-dithiothreitol (DTT; Roche, Basel, Switzerland) for 20 min at 65 °C, then alkylated (5.5 mM iodoacetamide; Sigma-Aldrich, St. Louis, MO, USA) for 30 min in darkness. An additional 10 mM DTT was added to samples, which were incubated for 30 min then digested overnight in proteomics-grade trypsin (Sigma-Aldrich, St. Louis, MO, USA) at 37 °C and quenched with 5% FA.

### 4.6. LC-ESI-MS/MS Tandem Mass Spectrometry

Tryptic digest samples were desalted with Millipore ZipTip^®^ C_18_ resin pipette tips (Millipore, Burlington, MA, USA) according to the manufacturer’s protocol, lyophilised and analysed in an Eksigent NanoLC Ultra (Eksigent, Framingham MA, USA) coupled with a SCIEX TripleTOF 5600 fitted with a NanoSpray III source (both SCIEX, Framingham, MA, USA). A total of 5 µL of each sample was injected into a C_18_ trap column (Eksigent ChromXP C18CL, 10 mm × 0.3 mm, 5-µm particle diameter, pore size 120 Å) (Eksigent, Framingham, MA, USA), desalted at 3 μL/min for 15 min with 100% Buffer A (0.1% FA) and separated in a C_18_ analytical column (Eksigent ChromXP C18CL, 0.075 mm × 150 mm, 3 µm particle diameter, pore size 120 Å) (Eksigent, Framingham, MA, USA) using a flow rate of 250 nL/min and a gradient of Buffer B (0.1% FA in ACN) as follows: 5–10% for 2 min; 10–40% over 58 min; 40–50% for 5 min; 50–95% for 10 min; 95% for 15 min; 95–5% for 4 min; and 5% for 6 min. MS data were acquired in positive TOF-MS mode using a scan range of 300–2000 Da with an accumulation time of 250 ms. Information-dependent acquisition was used to select precursor ions for MS/MS analysis with a charge state of 2^+^–5^+^ exceeding 50 counts per second. MS/MS data were acquired in positive product ion mode (high sensitivity), employing a scan range of 100–2000 Da. Both MS and MS/MS data were acquired with an ion spray voltage of 2.4 kV, a curtain gas flow of 25 psi, a nebulising gas flow of 5 psi, an interface heater temperature of 150 °C and a declustering potential of 80 V.

### 4.7. Protein Identification with ProteinPilot and MASCOT

MS/MS spectra from both pooled venoms were searched against the non-redundant NCBI Serpentes database (taxid: 8570) [54] and the UniProtKB Serpentes database [55]. The searches were conducted using ProteinPilot 4.0 with the Paragon algorithm (SCIEX, Framingham, MA, USA) and the MASCOT server accessed via MASCOT Daemon 2.5.1 (Matrix-Science, London, UK). ProteinPilot searches were conducted with a thorough search effort and a false discovery rate (FDR) analysis at ≥95% confidence, and matches were accepted only if the number of significant unique sequences ≥2. MASCOT search parameters allowed up to two missed trypsin cleavages, a 50-ppm peptide tolerance and a ±0.1-Da MS/MS tolerance. Carbamidomethylation of cysteine was set as a fixed modification and oxidation of methionine and deamidation of asparagine and glutamine as variable modifications. The FDR for peptide spectrum matches above homology was set to ≤1% and the number of significant unique sequences to ≥2.

### 4.8. Relative Protein Abundance Estimations

The MASCOT exponentially-modified protein abundance index, or emPAI protocol (Matrix-Science, London, UK), was employed to estimate the relative abundance of proteins in *H. curtus* venom by protein family. For each peak identified by RP-HPLC, the emPAI score of each matched protein was converted to a percentage of the total sum of the emPAI scores in the peak. The percentage each protein contributed to the peak area was calculated accordingly using the peak area from RP-HPLC chromatograms. Percentages were then added by protein family to compute the relative abundance of each family in the venom. Typical contaminants such as trypsin and keratin were removed.

### 4.9. Calculating Molecular Weights from Existing H. curtus Sequences

The ExPASy “compute pI/*M*w” tool (Swiss Institute of Bioinformatics, available online: web.expasy.org/compute_pi) was used to calculate the theoretical average molecular weight of amino acid sequences from proteomic and transcriptomic data found in the UniProtKB database [56]. The molecular weight was calculated from the chain sequence, excluding signal peptides and propeptides. Two mass units were subtracted from the calculated reduced peptide mass for every disulfide bond to obtain the oxidised peptide mass.

## Figures and Tables

**Table 1:** Assignment of RP-HPLC fractions by ESI-MS/MS of in-solution trypsin-digested peaks for *Hydrophis curtus* venom. Protein matches shown are from searches conducted with ProteinPilot and the Paragon algorithm against the UniProtKB Serpentes database for all peaks in the June pooled venom and from the peak marked with an asterisk (*) in the January pooled venom. Molecular weight (MW) data from ESI-MS analysis of June and January pooled venoms is listed in approximate order of descending intensity.

**Peak**	Peak Area %	June MW (Da)	January MW (Da)	Peptides (95%)	Score	Cov % (95%)	Protein Family	Related protein (exPASy MW)	Species (Accession)
**1**	10.1	6,688.5 6,745.3 6728.2	6,746.8 6,688.4 6,800.8	43	16.0	58.0	3FTx	Short neurotoxin 1 (6689.5)	*Hydrophis hardwickii* (P68416)
**2**	0.05	6,688.1 6,747.1 6,727.9 6,720.2 7,732.8 6,811.8	6,747.7 6,688.4 6,800.8 6,353.0 6,720.8 6,811.4 7,734.8	16	25.6	33.6	CRISP	Cysteine-rich venom protein 1	*Hydrophis hardwickii* (Q8UW25)
				8	13.5	52.2	3FTx	α-elapitoxin-Lh2a	*Hydrophis hardwickii* (Q8UW28)
				13	12.0	58.0	3FTx	Short neurotoxin 1 (6689.5)	*Hydrophis hardwickii* (P68416)
				6	10.2	58.1	3FTx	Long neurotoxin 2	*Hydrophis hardwickii* (A3FM53)
				16	9.1	28.6	CRISP	Cysteine-rich venom protein pseudechetoxin-like	*Notechis scutatus scutatus* (Q3SB04)
				17	2.0	29.4	CRISP	Cysteine-rich secretory protein	*Cryptophis nigrescens* (B5G6G3)
**3**	22.4	7,632.4 7,574.4 7,556.7 7,614.7	7,632.7 7,574.6 7,686.5 7,614.3 7,556.1	48	54.7	79.4	3FTx	Alpha-elapitoxin-Lh2a (7633.7)	*Hydrophis hardwickii* (Q8UW28)
				13	16.0	57.6	3FTx	Long neurotoxin 1	*Hydrophis hardwickii* (Q8UW29)
				4	6.4	43.2	3FTx	Short neurotoxin 1	*Hydrophis hardwickii* (P68416)
				9	6.0	58.1	3FTx	Long neurotoxin 2	*Hydrophis hardwickii* (A3FM53)
				4	4.2	13.5	CRISP	Cysteine-rich venom protein 2	*Hydrophis hardwickii* (Q8UW11)
				2	3.1	7.5	PLA_2_	Basic phospholipase A_2_	*Hydrophis hardwickii* (Q8UW08)
				8	2.2	20.3	3FTx	Toxin Lc a	*Laticauda colubrina* (P0C8R7)
**4**	4.5	13,252.0 13,307.7 13,325.1 7,632.3 7,574.6 15,670.2 7,733.5	13,336.1 13,308.8 7,632.6 13,328.7 7,574.1 13,251.7 13,389.7 7,614.6 7,686.5 15,726.2 15,672.7	113	93.9	77.4	PLA_2_	Basic phospholipase A_2_ 73 (13,310.0)	*Hydrophis hardwickii* (Q8UW30)
				42	31.2	79.4	3FTx	α-elapitoxin-Lh2a (7633.7)	*Hydrophis hardwickii* (Q8UW28)
				17	18.8	62.0	3FTx	Long neurotoxin 1	*Hydrophis hardwickii* (Q8UW29)
				61	12.2	74.7	PLA_2_	Basic phospholipase A_2_	*Hydrophis hardwickii* (Q8UW08)
				9	11.7	29.4	CRISP	Cysteine-rich venom protein 2	*Hydrophis hardwickii* (Q8UW11)
				4	7.9	58.0	3FTx	Short neurotoxin 1	*Hydrophis hardwickii* (P68416)
				14	4.2	29.0	PLA_2_	Acidic phospholipase A_2_ 57	*Hydrophis hardwickii* (Q8UW31)
				9	4.0	59.1	3FTx	Long neurotoxin 2	*Hydrophis hardwickii* (A3FM53)
				6	2.0	28.3	PLA_2_	PLA-2-Den-2	*Denisonia devisi* (R4G2S8)
**4–5**	–	–	–	154	86.9	76.7	PLA_2_	Basic phospholipase A_2_ 73	*Hydrophis hardwickii* (Q8UW30)
				117	21.4	75.3	PLA_2_	Basic phospholipase A_2_	*Hydrophis hardwickii* (Q8UW08)
				29	18.7	69.6	3FTx	α-elapitoxin-Lh2a	*Hydrophis hardwickii* (Q8UW28)
				15	12.3	62.0	3FTx	Long neurotoxin 1	*Hydrophis hardwickii* (Q8UW29)
				4	7.4	58.0	3FTx	Short neurotoxin 1	*Hydrophis hardwickii* (P68416)
				29	5.3	43.4	PLA_2_	Acidic phospholipase A_2_ 57	*Hydrophis hardwickii* (Q8UW31)
				7	3.2	12.6	CRISP	Cysteine-rich venom protein 2	*Hydrophis hardwickii* (Q8UW11)
				29	2.2	26.7	PLA_2_	Acidic phospholipase A_2_ S5-32M	*Austrelaps superbus* (Q9PUH4)
				3	2.1	4.2	SVMP	Nigrescease-1	*Cryptophis nigrescens* (B5KFV8)
				8	2.0	17.7	PLA_2_	Putative phospholipase A_2_	*Austrelaps labialis* (B2BRS9)
				4	2.0	20.3	3FTx	Toxin Lc b	*Laticauda colubrina* (P0C8R8)
**5**	3.3	13,344.9 13,401.7 7,632.5 7,574.4 13,269.8 13,364.8 13,326.8	13,344.7 13,401.4 7,632.4 7,574.1	116	122.7	76.0	PLA_2_	Basic phospholipase A_2_ (13,346.0)	*Hydrophis hardwickii* (Q8UW08)
				25	26.7	69.6	3FTx	α-elapitoxin-Lh2a (7633.7)	*Hydrophis hardwickii* (Q8UW28)
				20	26.0	60.5	PLA_2_	Acidic phospholipase A_2_ 57	*Hydrophis hardwickii* (Q8UW31)
				73	20.3	67.1	PLA_2_	Basic phospholipase A_2_ 73	*Hydrophis hardwickii* (Q8UW30)
				8	16.0	22.7	CRISP	Cysteine-rich venom protein 1	*Hydrophis hardwickii* (Q8UW25)
				9	12.1	15.2	SVMP	Zinc metalloproteinase-disintegrin-like MTP9	*Drysdalia coronoides* (F8RKV9)
				4	8.0	57.6	3FTx	Long neurotoxin 1	*Hydrophis hardwickii* (Q8UW29)
				3	6.0	43.2	3FTx	Short neurotoxin 1	*Hydrophis hardwickii* (P68416)
				16	4.0	34.8	PLA_2_	Basic phospholipase A_2_ 3	*Laticauda semifasciata* (P00612)
				2	2.0	20.3	3FTx	Toxin Lc a	*Laticauda colubrina* (P0C8R7)
*	2.5	–	14,157.2 7,632.1 13,401.6 13,345.2 7,574.6	144	136.6	77.6	PLA_2_	Acidic phospholipase A_2_ 57	*Hydrophis hardwickii* (Q8UW31)
				56	55.9	72.6	PLA_2_	Basic phospholipase A_2_ (13,346.0)	*Hydrophis hardwickii* (Q8UW08)
				19	18.6	64.1	3FTx	α-elapitoxin-Lh2a (7633.7)	*Hydrophis hardwickii* (Q8UW28)
				46	15.5	71.9	PLA_2_	Basic phospholipase A_2_ 73	*Hydrophis hardwickii* (Q8UW30)
				3	6.0	43.2	3FTx	Short neurotoxin 1	*Hydrophis hardwickii* (P68416)
				13	6.0	20.4	PLA_2_	PLA_2_-Bra-11	*Brachyurophis roperi* (R4G2E0)
				2	4.1	7.6	CRISP	Cysteine-rich venom protein 2	*Hydrophis hardwickii* (Q8UW11)
				2	2.7	20.3	3FTx	Toxin Lc a	*Laticauda colubrina* (P0C8R7)
				8	2.1	29.7	PLA_2_	Pa-18	*Pseudechis australis* (Q45Z21)
				9	2.0	17.1	PLA_2_	Acidic phospholipase A_2_ 2	*Ophiophagus hannah* (Q9DF33)
**6a, 6b, 6c**	47.9	14,215.4 14,314.8 7,632.6 14,269.3 7,574.8 13,113.8 13,402.4 13,170.5 7,685.9 13,325.3 7,615.0	14,215.5 14,314.6 7,632.4 7,574.3	116	115.2	73.7	PLA_2_	Acidic phospholipase A_2_ 57 (14,217.0)	*Hydrophis hardwickii* (Q8UW31)
				26	20.1	56.2	PLA_2_	Basic phospholipase A_2_	*Hydrophis hardwickii* (Q8UW08)
				8	14.0	64.1	3FTx	α-elapitoxin-Lh2a (7633.7)	*Hydrophis hardwickii* (Q8UW28)
				22	10.0	65.8	PLA_2_	Basic phospholipase A_2_ 73	*Hydrophis hardwickii* (Q8UW30)
				4	6.8	58.0	3FTx	Short neurotoxin 1	*Hydrophis hardwickii* (P68416)
				2	4.0	7.6	CRISP	Cysteine-rich venom protein 2	*Hydrophis hardwickii* (Q8UW11)
				12	2.1	47.7	PLA_2_	PLA-2	*Notechis scutatus* (Q45Z32)
				5	1.5	19.0	PLA_2_	Basic phospholipase A_2_ 2 (Fragment)	*Bungarus caeruleus* (Q6SLM1)
**7**	2.7	14,215.4 14,269.6 14,295.5 14,315.1 13,946.5	14,215.4 14,295.5 14,269.9 13,514.1 14,315.2 13,457.4	157	130.3	82.2	PLA_2_	Acidic phospholipase A_2_ 57 (14,217.0)	*Hydrophis hardwickii* (Q8UW31)
				17	10.3	56.9	PLA_2_	Basic phospholipase A_2_	*Hydrophis hardwickii* (Q8UW08)
				6	6.8	34.8	3FTx	α elapitoxin-Lh2a	*Hydrophis hardwickii* (Q8UW28)
				19	6.0	55.5	PLA_2_	Basic phospholipase A_2_ 73	*Hydrophis hardwickii* (Q8UW30)
				3	5.6	43.2	3FTx	Short neurotoxin 1	*Hydrophis hardwickii* (P68416)
				16	4.0	45.5	PLA_2_	Putative phospholipase A_2_	*Austrelaps labialis* (B2BRS7)
				9	4.0	45.2	PLA_2_	Acidic phospholipase A_2_ 2	*Ophiophagus hannah* (Q9DF33)
				4	3.2	12.2	CRISP	Cysteine-rich venom protein 1	*Hydrophis hardwickii* (Q8UW25)
				12	2.0	46.7	PLA_2_	PLA_2_ Hs-1	*Hoplocephalus stephensii* (A6MJG3)
**8a, 8b**	0.23	14,328.8 14,428.3 14,214.9 14,381.2 14,267.9	14,328.2 14,216.0 14,052.6 14,382.3 14,269.9	76	87.6	78.3	PLA_2_	Acidic phospholipase A_2_ 57 (14,217.0)	*Hydrophis hardwickii* (Q8UW31)
				16	16.4	56.9	PLA_2_	Basic phospholipase A_2_	*Hydrophis hardwickii* (Q8UW08)
				12	12.9	62.0	3FTx	α-elapitoxin-Lh2a	*Hydrophis hardwickii* (Q8UW28)
				4	6.6	16.8	CRISP	Cysteine-rich venom protein 1	*Hydrophis hardwickii* (Q8UW25)
				3	6.4	43.2	3FTx	Short neurotoxin 1	*Hydrophis hardwickii* (P68416)
				18	6.0	55.5	PLA_2_	Basic phospholipase A_2_ 73	*Hydrophis hardwickii* (Q8UW30)
				6	4.0	31.8	PLA_2_	PLA-2	*Notechis scutatus* (Q45Z32)
**9a**	0.7	15,045.6 15,144.9 14,216.4 14,975.0 15,102.3 14,847.0 14,934.0 15,003.3 15,074.1	15,046.2 15,005.0 15,144.2 15,101.5 14,846.2 14,975.0 14,886.8 15,370.2	64	65.6	80.9	PLA_2_	Acidic phospholipase A_2_ 57 (14,217.0)	*Hydrophis hardwickii* (Q8UW31)
				17	12.6	73.3	PLA_2_	Basic phospholipase A_2_	*Hydrophis hardwickii* (Q8UW08)
				8	7.8	52.2	3FTx	α-elapitoxin-Lh2a	*Hydrophis hardwickii* (Q8UW28)
				18	6.0	61.0	PLA_2_	Basic phospholipase A_2_ 73	*Hydrophis hardwickii* (Q8UW30)
				3	5.6	43.2	3FTx	Short neurotoxin 1	*Hydrophis hardwickii* (P68416)
				7	3.1	27.3	CRISP	Cysteine-rich venom protein 1	*Hydrophis hardwickii* (Q8UW25)
**9b**	0.5	15,044.9 15,004.8 14,845.6 14,934.0 14,215.6 20,615.9	15,045.6 15,004.0 14,846.8 14,214.6 14,933.4 15,099.5 14,973.8 15,145.5 15,073.4 14,886.3 20,614.6	103	99.3	88.2	PLA_2_	Acidic phospholipase A_2_ 57 (14,217.0)	*Hydrophis hardwickii* (Q8UW31)
				20	16.5	47.1	CRISP	Cysteine-rich venom protein 1	*Hydrophis hardwickii* (Q8UW25)
				16	13.6	59.0	PLA_2_	Basic phospholipase A_2_	*Hydrophis hardwickii* (Q8UW08)
				6	6.9	24.3	PEBP	Phosphatidylethanolamine-binding protein 4	*Micrurus fulvius* (U3FZ77)
				17	5.6	61.0	PLA_2_	Basic phospholipase A_2_ 73	*Hydrophis hardwickii* (Q8UW30)
				4	5.4	58.0	3FTx	Short neurotoxin 1	*Hydrophis hardwickii* (P68416)
				5	5.0	51.1	3FTx	α-elapitoxin-Lh2a	*Hydrophis hardwickii* (Q8UW28)
				2	1.7	3.8	Hsp70	78 kDa glucose-regulated protein	*Ophiophagus hannah* (V8NEC1)
				16	1.4	37.0	CRISP	Cysteine-rich venom protein 2	*Hydrophis hardwickii* (Q8UW11)
**10**	7.5	24,321.2 24,420.2 24,223.2 24,521.0 24,058.7 14,215.2	24,320.5 24,419.6 24,520.7 24,222.4 24,058.3 14,215.2	86	77.4	75.2	CRISP	Cysteine-rich venom protein 2 (24,522.8)	*Hydrophis hardwickii* (Q8UW11)
				12	9.4	68.4	PLA_2_	Acidic phospholipase A_2_ 57 (14,217.0)	*Hydrophis hardwickii* (Q8UW31)
				6	7.6	37.7	PLA_2_	Basic phospholipase A_2_	*Hydrophis hardwickii* (Q8UW08)
				4	5.7	28.4	Cys	Cystatin	*Hoplocephalus stephensii* (E3P6P0)
				3	5.4	43.2	3FTx	Short neurotoxin 1	*Hydrophis hardwickii* (P68416)
				50	5.3	63.0	CRISP	Cysteine-rich venom protein 1 (24,334.8)	*Hydrophis hardwickii* (Q8UW25)
				23	4.9	36.1	CRISP	Cysteine-rich venom protein pseudechetoxin	*Pseudechis australis* (Q8AVA4)
				7	4.7	47.3	PLA_2_	Basic phospholipase A_2_ 73	*Hydrophis hardwickii* (Q8UW30)
				6	2.7	52.2	3FTx	α-elapitoxin-Lh2a	*Hydrophis hardwickii* (Q8UW28)
**11**	0.13	43,732.5	43,730.9 43,414.4	32	51.2	60.1	CRISP	Cysteine-rich venom protein 2	*Hydrophis hardwickii* (Q8UW11)
				13	26.1	67.1	PLA_2_	Acidic phospholipase A_2_ 57	*Hydrophis hardwickii* (Q8UW31)
				30	14.5	52.5	CRISP	Cysteine-rich venom protein 1	*Hydrophis hardwickii* (Q8UW25)
				9	12.6	45.2	PLA_2_	Basic phospholipase A_2_	*Hydrophis hardwickii* (Q8UW08)
				5	10.7	5.0	Golgin	Golgi apparatus protein 1 (Fragment)	*Ophiophagus hannah* (V8NSK8)
				5	10.7	5.8	Trans	Transferrin	*Boiga irregularis* (A0A0B8RPR5)
				5	7.8	51.1	3FTx	α-elapitoxin-Lh2a	*Hydrophis hardwickii* (Q8UW28)
				3	6.1	43.2	3FTx	Short neurotoxin 1	*Hydrophis hardwickii* (P68416)
				10	6.0	52.7	PLA_2_	Basic phospholipase A_2_ 73	*Hydrophis hardwickii* (Q8UW30)
				5	6.0	10.5	Nucleo	Nucleobindin-2	*Micrurus fulvius* (U3FBZ2)
				2	4.2	11.6	CTL	C-type lectin 1	*Hydrophis hardwickii* (A3FM55)
				2	4.1	7.6	PC1A	Cathepsin B	*Micrurus fulvius* (U3FD65)
				12	4.0	30.7	CRISP	Cysteine-rich venom protein pseudechetoxin	*Pseudechis australis* (Q8AVA4)
				3	2.2	6.0	Trans	Transferrin	*Boaedon fuliginosus* (Q1EL74)

The unique January peak is labelled with an asterisk (*). Peak areas are all calculated from the RP-HPLC chromatogram of June pooled venom, except for the unique January peak, which is from the RP-HPLC chromatogram of January pooled venom. ESI-MS molecular weights (MW in Da) are listed in approximate descending order of intensity. Molecular weights in bold blue are similar in size to known *H. curtus* proteins, as calculated by exPASy. Average exPASy calculated molecular weights (exPASy MW) are in brackets after related proteins where a similar molecular weight was detected by ESI-MS in the same peak. Unused score values and the number of peptides and coverage (Cov) with ≥95% confidence are calculated with the Paragon algorithm of ProteinPilot. Protein family abbreviations: 3FTx = three-finger toxin; CRISP = cysteine-rich secretory protein; PLA_2_ = phospholipase A_2_; SVMP = snake venom metalloproteinase; PEBP = phosphatidylethanolamine-binding protein; Hsp70 = heat shock protein; Cys = cystatin; Golgin = golgin; Trans = transferrin; Nucleo = nucleobindin; CTL = C-type lectin; PC1A = Peptidase C1A. Cysteine residues are carbamidomethylated. Full details of MS/MS-derived sequences and the masses and charges of peptide ions for ProteinPilot searches can be found in the Supplementary Material (Supplementary Tables S1–S4).

## Supplementary Materials

Supplementary materials can be found at www.mdpi.com/1422-0067/18/12/2695/s1.

## Abbreviations

3FTx	Three-Finger Toxin
ACN	Acetonitrile
CRISP	Cysteine-Rich Secretory Protein
CTL	C-Type Lectin
Cys	Cystatin
DTT	1,4-Dithiothreitol
ECM1	Extra-Cellular Matrix Protein
FA	Formic Acid
FDR	False Discovery Rate
Hsp70	Heat Shock Protein
LC-ESI-MS	Liquid Chromatography Electrospray Ionisation Mass Spectrometry
MALDI-TOF	Matrix-Assisted Laser Desorption Ionisation-Time of Flight
MQ	Milli-Q
nAChR	Nicotinic Acetylcholine Receptor
Nucleo	Nucleobindin
PC1A	Peptidase C1A
PEBP	Phosphatidylethanolamine-Binding Protein
PLA_2_	Phospholipase A_2_
RNase T2	Ribonuclease T2
RP-HPLC	Reversed-Phase High-Performance Liquid Chromatography
SVMP	Snake Venom Metalloproteinase
SVSP	Snake Venom Serine Protease
TFA	Trifluoroacetic Acid
Trans	Transferrin
