# Supplementary material for: The Venom of the Spine-Bellied Sea Snake (Hydrophis curtus): Proteome, Toxin Diversity and Intraspecific Variation
Source: Int J Mol Sci. 2017 Dec 12;18(12):2695. doi: 10.3390/ijms18122695 (PMC5751296; doi:10.3390/ijms18122695)
Supplement: Supplementary file 1 [file ijms-18-02695-s001.zip › Supplementary materials S9, Figure S1.docx]

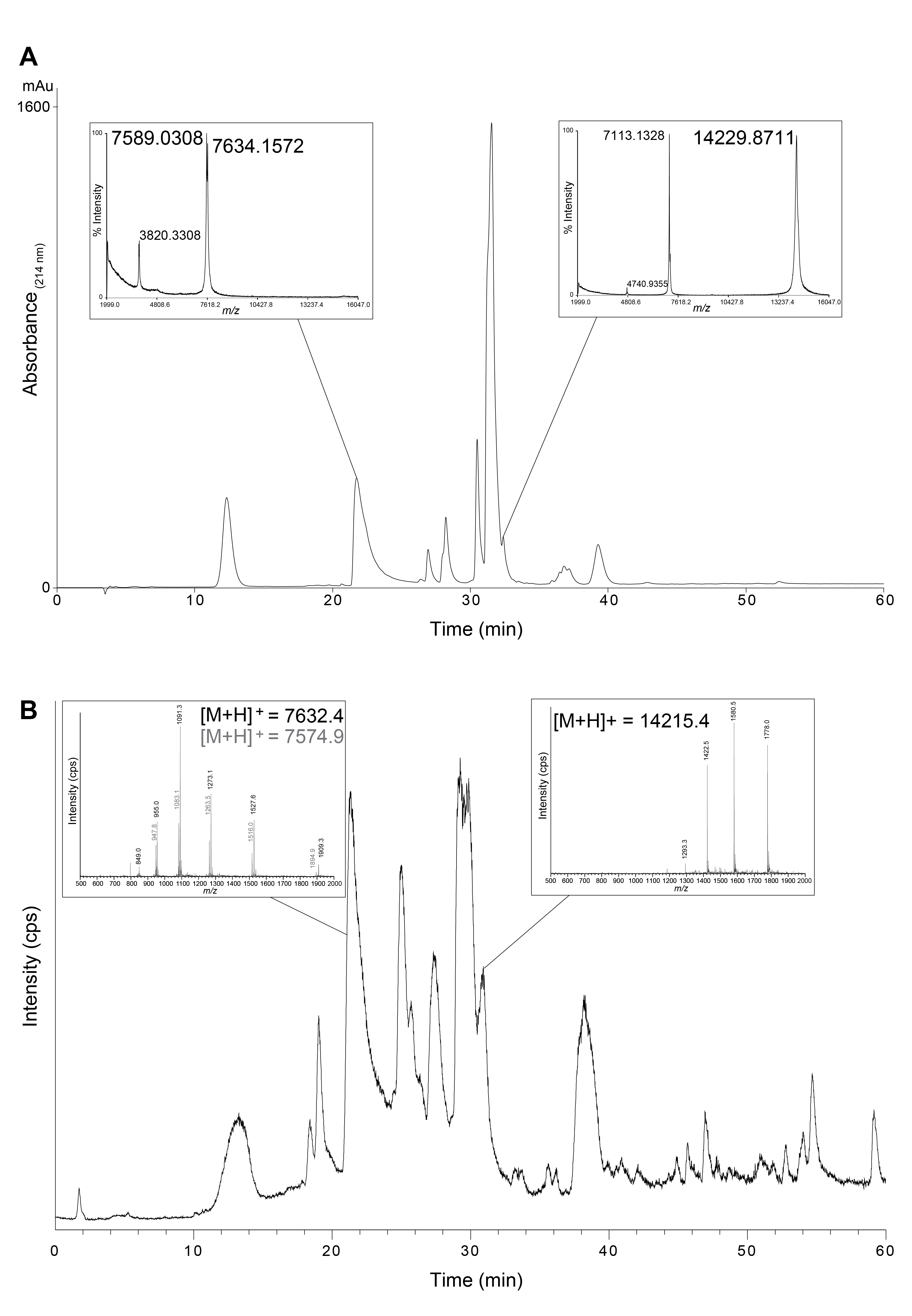


**Figure S1.** Representative spectra from two peaks of *Hydrophis curtus* venom by MALDI-TOF and ESI-MS, demonstrating consistency between the two methods. (**A**) shows the RP-HPLC chromatogram (Phenomenex Jupiter C_4_, 4.6 × 250 mm, 5 µm, 300 Å) (Phenomenex, Torrance, CA, USA), with the solvent gradient 0−60% solvent B over 60 min) of *H. curtus* sample 11 from January 2016 with MALDI-TOF (SCIEX TOF/TOF™ 5800 MALDI mass spectrometer, SCIEX, Framingham, MA, USA) linear positive mode spectra inset. The matrix used was α-cyano-4-hydroxycinnamic acid. (**B**) shows the total ion current chromatogram of an LC-ESI-MS run (Shimadzu LC-MS 2020, Shimadzu, Kyoto, Japan with Phenomenex Aeris XB-C_18_ column, 2.1 × 150 mm, 3.6 µm, 100 Å (Phenomenex, Torrance, CA, USA), using a solvent gradient of 0−60% solvent B over 60 min, mass range scan *m/z* 250-2000) on June pooled venom with examples of underlying spectra inset.
